# Impact of controlled release urea on maize yield and nitrogen use efficiency under different water conditions

**DOI:** 10.1371/journal.pone.0181774

**Published:** 2017-07-24

**Authors:** Guanghao Li, Bin Zhao, Shuting Dong, Jiwang Zhang, Peng Liu, Tony J. Vyn

**Affiliations:** 1 State Key Laboratory of Crop Biology and College of Agronomy, Shandong Agricultural University, Tai’an, Shandong, PR China; 2 Agronomy Department, Purdue University, West Lafayette, Indiana, United States of America; Shandong University, CHINA

## Abstract

Controlled release urea (CRU) has been widely adopted to increase nitrogen (N) use efficiency and maize production, but the impacts can range widely depending on water availability in the soil. In an experiment using Zhengdan 958 (a popular summer maize hybrid), three levels of water treatments (adequate water condition [W3], which maintained soil moisture at about 75% ± 5% of the soil’s field capacity; mild water stress [W2], which maintained moisture content at 55% ± 5% of field capacity; and severe water stress [W1], which had a moisture content of 35% ± 5% of field capacity) and four levels of controlled release urea fertilizer (N0, N1, N2 and N3 were 0, 105, 210 and 315 kg N ha^–1^, respectively) were compared in a rainout shelter system with soil. The results revealed that CRU had significant effects on maize yields and N use efficiencies under different water conditions. The mean yields increased with increasing water levels and showed significant differences. Under W1, the accumulation of dry matter and N were limited, and N internal efficiency (NIE) and the apparent recovery efficiency of applied N (RE_N_) decreased with N increases; yields of N1, N2, and N3 were similar. Under W2, the dry matter and N accumulation, as well as the yield, showed an increasing trend with an increase in N application, and the NIE and RE_N_ of N3 showed no difference from N2. Under W3, yields of N2 and N3 were similar and they were significantly higher than that of N1, but the agronomic N use efficiency (ANUE), RE_N_, and the physiological NUE (PNUE) of N2 were 54.2, 34.9, and 14.4% higher, respectively, than those of N3. N application beyond the optimal N rate did not consistently increase maize yield, and caused a decrease in N use efficiencies. Highest overall dry matter, N accumulation, and yields were observed with N3 under W2, and those showed no differences with N2 and N3 under W3. Under this experimental condition, the CRU of 210 kg ha^–1^ was optimized when soil moisture content was 75% ± 5% of field capacity, but an N rate of 315 kg ha^–1^ was superior when soil moisture content during the entire growing season was maintained at 55% ± 5% of field capacity.

## Introduction

Maize (*Zea mays* L.) is a major grain crop in China. With the development of national green agriculture, maize is playing an increasingly important role in crop production and the pressure of increasing maize production in the future is high [1]. Summer maize production in China is challenging due to both drought stress and low nitrogen (N) use efficiency (NUE) [2]. Water is one of the most important limiting factors for maize production on available arable lands [3]. Maize grown in northern China is often influenced by inadequate soil water, and drought stress, and whether early or later on in the growing season, this can substantially reduce crop yield [4, 5]. Therefore, effective water management for agricultural production in water-scarce regions requires the application of innovative and sustainable approaches. Currently, adopting superior maize cultivars and increasing fertilizer inputs have contributed to enhanced total production capacity [6, 7]. N fertilizers play an essential role in our modern agricultural production systems. The actual average N utilization rate in the Huanghuaihai plain was 276 kg ha^–1^, which was significantly higher than that of China by 220 kg ha^–1^ [2, 8]. High N fertilization has resulted in low NUE and in nitrate ground-water contamination, which not only wastes resources and energy [9] but seriously affects the agricultural and ecological environment [2, 10]. Due to economic factors and environmental concerns, balancing fertilizer input and output has received a lot of attention. Some studies have suggested that using multiple in-season N fertilizer applications instead of a single pre-plant application can increase aboveground dry matter yield of summer maize, thereby also improving the utilization rate of N [11–14]. However, such multiple N applications would undoubtedly increase labor costs regardless of the type of N fertilizer source or placement employed in China.

Research on the application of slow-release fertilizer as an alternative to multiple N application timings has been pursued internationally [15]. The N release rate of slow-release fertilizer corresponds more closely to crop plant N requirements for physiological functions [16]. Its one-time application is also more convenient than multiple N applications, so this method reduces labor costs since fewer N applications are required. In addition to reducing labor inputs, slow-release fertilizer can effectively increase the utilization rate of N and production efficiency [17, 18]. There are a large number of studies showing that slow/controlled release fertilizer can improve NUE and maize yield [19, 20].

Previous reports have demonstrated that N fertilizer management [11, 21] and water management [4, 22] are closely linked and have significant interaction effects [23–27] that extend to N accumulation, translocation, and partitioning. Therefore, the objective of this study was to determine the interactive effects of water and controlled release urea on yield, dry matter accumulation, and N absorption and distribution in summer maize after the tasseling stages. The study was specifically designed to focus on the impacts of controlled release urea on increasing maize yield and improving NUE under different water stress conditions. This study may provide information about optimum production management techniques for controlled release urea fertilizer as it relates to water conservation and the production of high yields.

## Materials and methods

### Plant materials and experimental location

This study was conducted at the Maize Technological Innovation Center in the Huanghuaihai plain and the State Key laboratory of Crop Biology, located at Shandong Agriculture University (36°10′19′′N, 117°9′03′′E, 128 m above sea level) in Tai’an, China in 2013 and 2014. The region is characterized by a temperate continental monsoon climate. The mean effective accumulated temperature of summer maize growth periods during 2013 and 2014 were 1673°Cd and 1741°Cd, respectively. The mean total amounts of precipitation that occurred during summer maize growth periods in 2013 and 2014 were 401.3 mm and 366.0 mm, respectively. We used the summer maize hybrid Zhengdan 958 (released in 2000), which is widely grown in China. The estimated duration of the crop season of the hybrid was about 114 days. The experimental soil type was brown loam and its pH was 6.1. The average content of organic matter in the tillage layer (0–40 cm) was 11.3 g kg^–1^ and the available N, available phosphorous (P) and exchangeable potassium (K) were 124 mg kg^–1^, 45 mg kg^–1^, and 82 mg kg^–1^, respectively. The soil moisture content at field capacity was 21% and the mean bulk density was 1.5 g cm^–3^. The soil pH value was determined by a pH-meter in the soil, and the water ratio was 1:2.5. Organic matter was determined using a titration method after digestion with a K_2_Cr_2_O_7_-H_2_SO_4_ solution. Available P was extracted with 0.5 mol L^–1^ NaHCO_3_ and determined using colorimetric analysis. Exchangeable K was extracted with 1 mol L^–1^ NH_4_OAc and then determined using the atomic absorption spectrometry method [28]. The soil field capacity and bulk density were determined with the Welcox method [29]. The study made use of pot experiments (with clay pots that were 35 cm in diameter and 45 cm high). The 40-cm pots were filled with brown loam soil, which was taken from normal farmland. The soils were divided into two layers and each layer was 20 cm thick. The 20-cm of soil from each layer was blended after sieving and then backfilled into the corresponding layer of the pots.

### Experimental design

There were three levels of water treatment (adequate water conditions [W3], which kept the soil moisture at about 75% ± 5% of the soil’s field capacity; mild water stress [W2], which maintained a moisture content of 55% ± 5% of field capacity; and severe water stress [W1], which had a moisture content of 35% ± 5% of the soil’s field capacity). Four levels of controlled release urea (CRU) fertilizer (N0 was no N, N1 was 105 kg N ha^–1^, N2 was 210 kg N ha^–1^, and N3 was 315 kg N ha^–1^) were applied to all three water treatments. The amount of N3 fertilizer was the same as that used by traditional farmers. Zhao et al. [30] demonstrated that maize yield could be guaranteed by reducing controlled release urea properly. So we used four gradient N applied amounts to determine the interactive impact of CRU under severe, mild water stress and adequate water condition. All of the CRU was applied at sowing. The CRU fertilizer (coated with a resin polymer, with an N content of 42%, made by Kingenta Ecological Engineering Co., Ltd., Shandong, China) was used for crops. N release longevity of the CRU in water at a temperature of 25°C was about three months, and could be basically consistent with the maize nutrient requirement during the whole growth period [31]. All treatments received the same 105 kg ha^–1^ P_2_O_5_ (P_2_O_5_ 1.56 g per pot) and 210 kg ha^–1^ K_2_O (K_2_O 3.12 g per pot) as a basal dressing. There were 12 treatment-combinations (water regime × N rates) in this experiment and each treatment had 30 pots, which was together a total of 360 pots. Each treatment had two close rows and 15 pots per row, and each sampling was sequentially taken along the rows. Maize was sown on June 18 and harvested on October 1 in both 2013 and 2014. Plants were grown outdoors. Four seeds were planted in each pot. At the third-leaf stage, three plants were thinned and the remaining plant was allowed to grow in the pot until harvest. Two rows of summer maize were planted all around the experimental area as borders. During the pre-sowing period, irrigation was applied to field capacity to ensure full stand establishment in all treatments. Soil moisture was measured randomly for five pots using a TDR meter (Delta UK Ltd., Clacton-on-Sea, UK) in the morning and evening of each day. According to the measured soil water content, soil bulk density, soil moisture maximum field capacity and soil weight, the amount of needed water under different water conditions was calculated. Water stress treatments were imposed when soil moisture first fell below the designated standards during the summer maize season. Automatically operated, triple-folding rain-shelters were moved over the test before rainfall, to prevent natural rainfall over pots.

### Plant sampling and N content determination

Three representative plant samples were obtained in each treatment from the tasseling stage (when each treatment reached 50% of tassel emergence) to the physiological maturity stage (R6) at approximately 12-day intervals. Samples were separated into sheath, stalk, leaf, tassel, bract, cob, and grain sections. They were then dried at 80°C in a forced-air oven (DHG-9420A; Shanghai Bilon Instruments Co. Ltd., Shanghai, China) to a constant weight and weighed separately. After weighing, the samples were grounded using a cyclone sample mill with a fine mesh (0.5 mm). The N concentrations of different organs were then measured using the micro-Kjeldahl method (CN61M/KDY-9820; Beijing, China). The following parameters [12, 32–34] were calculated:

$$\text{Plant N uptake }\left({\text{g plant}}^{-1}\right)\;=\text{ the plant N concentration}\times \text{dry matter weight of plant}$$

Nitrogen harvest index (NHI, %)=Grain N / (Shoot N + Grain N)

where the shoot N fraction includes stem, leaf, cob, bract, and sheath components, and the grain N fraction is only composed of the grain component.

$$\begin{array}{l} \text{Nitrogen internal efficiency }\left(\text{NIE},{\text{ kg kg}}^{-1}\right)\;=\text{ grain weight }/(\text{N weight in the whole-plant at maturity which is the plant DM multiplied by the plant N concentration}) \end{array}$$

$$\text{Agronomic NUE }\left(\text{ANUE},{\text{ kg kg}}^{-1}\right)\;=\;[\text{grain weight }(\text{with fertilizer})-\text{grain weight }\left(\text{no fertilizer}\right)]\;/\text{ N fertilizer applied}$$

$$\text{Apparent recovery efficiency of applied N }\left({\text{RE}}_{\text{N}},\;\%\right)\;=\;\left[{\text{N uptake at N}}_{\text{x}}-{\text{ N uptake at N}}_{\text{0}}\right]\;/{\text{ applied N at N}}_{\text{x}}\times \;100$$

$$\text{Physiological NUE }\left(\text{PNUE},{\text{ kg kg}}^{-1}\right)\;=\;\left[\text{grain weight }\left(\text{fertilizer}\right)-\text{grain weight }\left(\text{no fertilizer}\right)\right]\;/\;\left[\text{plant N }\left(\text{fertilizer}\right)-\text{plant N }\left(\text{no fertilizer}\right)\right]$$

Soil nitrogen dependency ratio (SNDR, %) = plant N content(no fertilizer) / plant N content (with fertilizer)× 100

### Yield

At the physiological maturity stage (R6), all of the remaining ears were harvested to determine yield (moisture content was approximately 14%) and ear traits.

$$\text{Grain yield per plant }\left({\text{g plant}}^{-1}\right)\;=\text{ grain number per ear }\times \;1000\text{-grain weight }\left(\text{g}/1000\text{ grains}\right)\;/\;1000\;\times \;\left(1-\text{moisture content}\right)/\;\left(1-14\%\right)$$

### Statistical analysis

The data were subjected to two-way analysis of variance (ANOVA). Growing season, blocks, and block interactions were included as random effects. Water and N were included as fixed effects. In the case of significant treatment effects, comparison of means was performed using the LSD at a significance level of 0.05. The LSD was used to compare adjacent means arranged in order of magnitude. ANOVA and the LSD test were conducted using the SPSS17.0 program (Ver. 17.0, SPSS, Chicago, IL, USA). Results were presented as means of the two years of experimentation except for grain yield, because the trends of these parameters were consistent between the two years. Calculations and linear regressions were performed using the SigmaPlot 10.0 program.

## Results

### Yield and yield components

Yield was affected by water, controlled release urea (CRU), and their interaction for both years (P < 0.01). Their interactions on grains per ear and 1000-grain weight were very significant (P < 0.01) (Table 1). Mean yields increased with increasing water levels and showed significant differences. Under W1, the yields of N1, N2, and N3 were similar and they were significantly higher than that of N0. Under W2, yields showed an increasing trend with increased amounts of N application; yield of N3 was 32.5, 16.3, and 12.7% higher than N0, N1, and N2 respectively in 2013, and 25.7, 17.7, and 22.2% higher in 2014; yields of N1 and N2 were similar and grains per ear and 1000-grain weight were consistent with yields. Under W3, yields of N2 and N3 showed no significant differences in both years. However, they were significantly higher than those of N0 and N1. The yield of N3 was the highest of all the N treatments only under W2 conditions in 2013 and 2014 (Table 1).

**Table 1:** Impact of controlled release urea (N0: no nitrogen, N1: N application of 105 kg hm^-2^, N2: N application of 210 kg hm^-2^, N3: N application of 315 kg hm^-2^) on yield and yield components of summer maize under different water conditions (W1: severe water stress, W2: mild water stress, W3: adequate water condition).

Water (W)	Nitrogen (N)	2013			2014		
		Grains per ear	1000-grain weight (g)	Yield per plant (g)	Grains per ear	1000-grain weight (g)	Yield per plant (g)
W1	N0	294.5±10.9g	277.7±10.0c	76.9±5.6f	348.2±9.9g	270.3±11.2e	85.7±7.0f
	N1	378.0±11.0f	253.1±5.5d	87.0±6.5e	393.5±12.9f	282.3±9.7de	105.3±8.0e
	N2	427.5±10.5e	244.3±5.0e	88.4±5.5e	402.6±8.0f	284.9±12.0d	106.8±6.6e
	N3	416.0±10.1e	254.8±5.5d	93.0±9.9de	420.2±12.2e	302.5±13.4c	113.3±4.1de
W2	N0	423.0±8.3e	277.2±9.1c	104.7±5.5d	425.3±7.8e	285.2±7.1d	129.8±3.2d
	N1	455.9±8.6d	310.3±9.2a	119.3±5.5c	459.4±6.8d	311.6±8.0bc	138.6±6.5c
	N2	468.0±10.5cd	314.8±9.0a	123.1±6.6c	480.2±6.5c	321.8±6.5ab	133.5±6.1c
	N3	561.2±8.2a	294.8±11.3b	138.7±2.6ab	563.2±7.9a	323.4±11.8ab	163.2±6.6ab
W3	N0	480.0±10.0c	291.6±5.2b	118.8±8.6c	488.2±5.5c	302.8±13.6c	133.8±5.3c
	N1	512.9±13.9b	295.6±4.8b	130.9±6.3b	537.9±8.2b	320.2±11.4ab	158.6±5.6b
	N2	576.7±5.8a	315.5±7.7a	144.7±6.1a	570.3±5.9a	330.8±10.4a	170.9±9.6a
	N3	560.0±11.5a	321.7±7.7a	144.0±6.0a	566.8±8.0a	325.7±4.6a	167.0±9.0a
Average							
W1		379.0±60.2c	257.5±14.2b	86.3±6.8c	391.1±30.7c	285.0±13.3b	103.1±12.4c
W2		477.0±59.3b	299.3±17.0a	121.5±14.0b	482.0±58.7b	310.5±17.6a	131.5±19.5b
W3		532.4±44.2a	306.1±14.7a	134.6±12.3a	540.8±38.0a	319.9±12.2a	153.4±17.3a
ANOVA							
W		**	*	**	**	**	**
N		*	NS	**	**	*	**
W×N		**	**	**	**	**	**

In each data area, different letters within the same column indicate significant difference among treatments at P<0.05. NS means not significant,

* and ** indicate significant difference at the 0.05 and 0.01 levels of probability, respectively.

### Plant dry matter accumulation and grain dry weight

**Fig 1:**
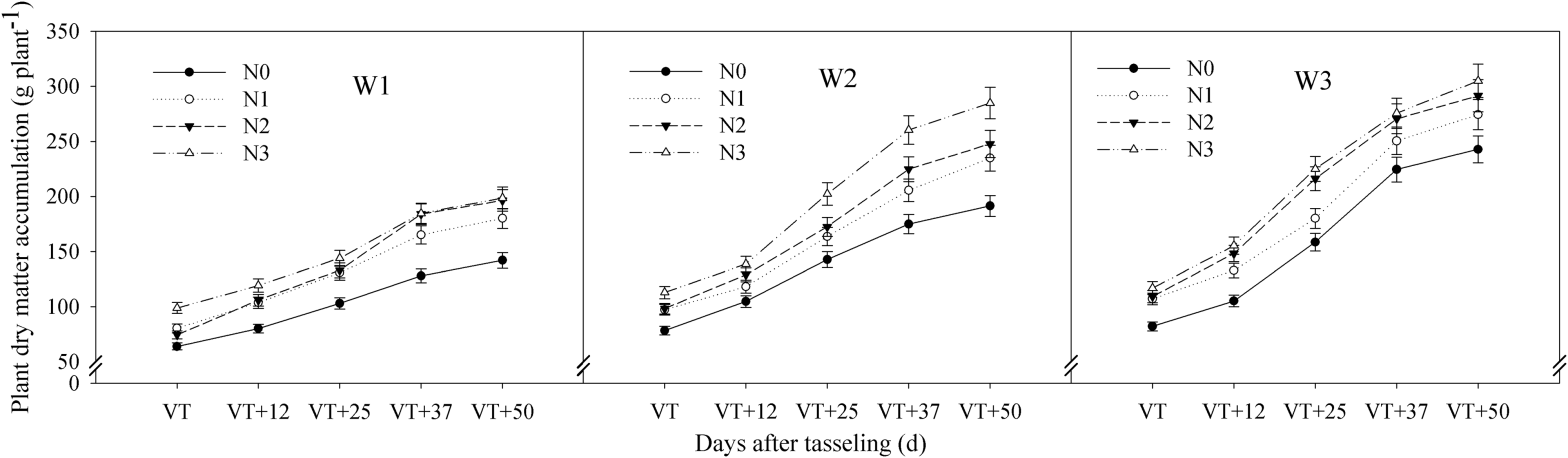
Impact of controlled release urea on plant dry matter accumulation of summer maize under different water conditions. W1: severe water stress; W2: mild water stress; W3: adequate water condition; N0: no nitrogen; N1: N application of 105 kg hm^-2^; N2: N application of 210 kg hm^-2^; N3: N application of 315 kg hm^-2^; VT: tasseling stage.

**Fig 2:**
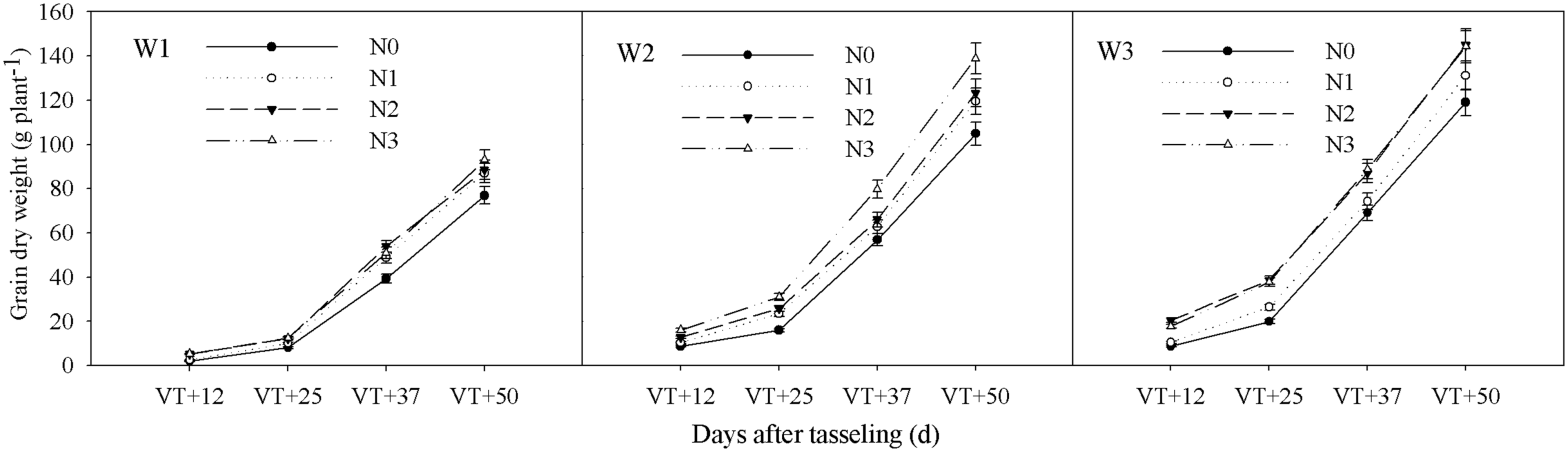
Impact of controlled release urea on grain dry weight of summer maize under different water conditions. W1: severe water stress; W2: mild water stress; W3: adequate water condition; N0: no nitrogen; N1: N application of 105 kg hm^-2^; N2: N application of 210 kg hm^-2^; N3: N application of 315 kg hm^-2^; VT: tasseling stage.

The interactive effects of water and CRU on plant dry matter accumulation (DM) and grain dry weight of summer maize met the extremely significant level (P < 0.01) (Tables 2 and 3). The DM under each treatment increased gradually after the tasseling stage and reached a maximum at maturity (Fig 1). At all of the growth stages, the average DM differences under three water conditions showed that W3 > W2 > W1, and the differences were significant (Table 2). Under W1, the DMs were relatively low, though they increased with an increasing amount of N fertilizer. Under W2, the DM rate at the tasseling stage was relatively high and the DM rates of N1, N2, and N3 were significantly higher than that of N0. Under W3, the DM in each treatment increased quickly, especially from 12 to 37 days after the tasseling stage (Fig 1). The grain dry weight of summer maize increased slowly during the early growth stage, relatively faster at the late growth stage, and reached a maximum at maturity (Fig 2). The dynamic changes in grain dry weight were consistent with the changes in total dry matter. The average grain dry weight also showed W3 > W2 > W1 after the tasseling stage (Table 3). Under W1, grain dry weights of each treatment increased slowly, and N3 and N2 were similar, but they were significantly higher than those of N1 and N0. Under W2, the grain dry weight increased relatively quickly, and the growth rate of N3 was the fastest. Under W3, the grain dry weight in each treatment increased quickly, particularly within the first 12 days after the tasseling stage (Fig 2).

**Table 2:** Analysis of variance for controlled release urea and water on total dry matter accumulation of summer maize after tasseling stages (VT).

Average	Days after tasseling (d)				
	VT	VT+12	VT+25	VT+37	VT+50
W1	79.4±14.6c	102.2±16.2c	127.7±17.5c	165.4±26.6c	179.3±26.1c
W2	96.6±14.2b	122.6±14.5b	170.2±24.7b	216.3±35.8b	239.6±38.5b
W3	103.8±15.0a	135.3±22.2a	194.9±31.1a	255.1±23.2a	278.2±26.8a
ANOVA					
W	**	**	**	**	**
N	NS	*	**	**	**
W×N	**	**	**	**	**

NS means not significant,

* and ** indicate significant difference at the 0.05 and 0.01 levels of probability, respectively.

**Table 3:** Analysis of variance for controlled release urea and water on grain dry weight of summer maize after tasseling stages (VT).

Average	Days after tasseling (d)			
	VT+12	VT+25	VT+37	VT+50
W1	3.8±1.7b	10.7±2.0b	48.3±6.3c	86.3±6.8b
W2	11.8±3.1a	23.8±6.3a	66.2±9.7b	121.5±14.0a
W3	14.2±5.6a	30.5±9.0a	79.6±9.7a	134.6±12.3a
AVOVA				
W	**	**	**	**
N	NS	NS	**	*
W×N	**	**	**	**

NS means not significant,

* and ** indicate significant difference at the 0.05 and 0.01 levels of probability, respectively.

### Changes in N accumulation

**Fig 3:**
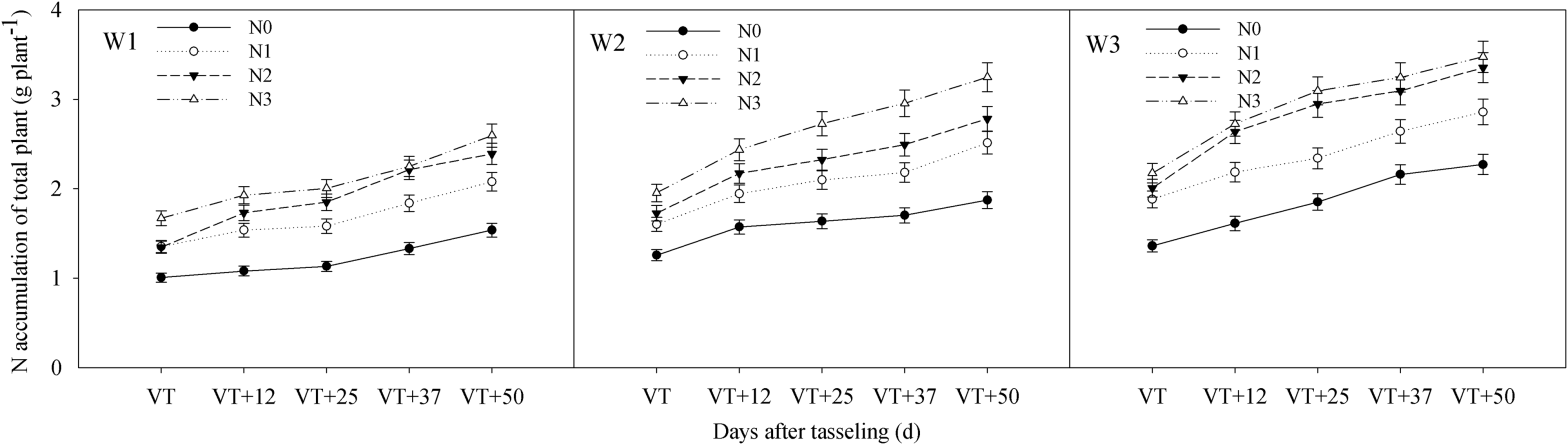
Impact of controlled release urea on total plant N accumulation of summer maize under different water conditions. W1: severe water stress; W2: mild water stress; W3: adequate water condition; N0: no nitrogen; N1: N application of 105 kg hm^-2^; N2: N application of 210 kg hm^-2^; N3: N application of 315 kg hm^-2^; VT: tasseling stage.

**Fig 4:**
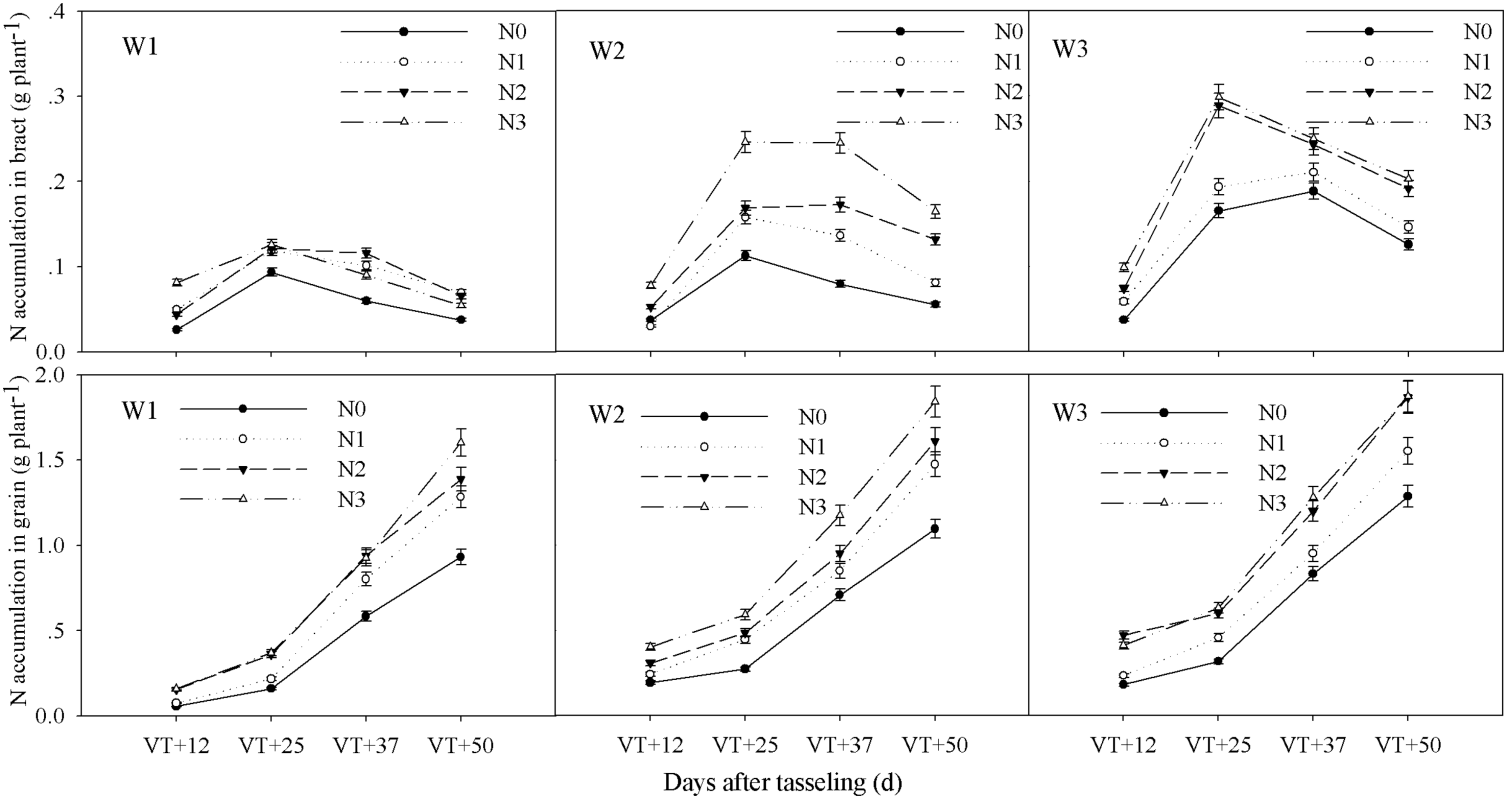
Impact of controlled release urea on N accumulation in bract and grain of summer maize under different water conditions. W1: severe water stress; W2: mild water stress; W3: adequate water condition; N0: no nitrogen; N1: N application of 105 kg hm^-2^; N2: N application of 210 kg hm^-2^; N3: N application of 315 kg hm^-2^; VT: tasseling stage.

**Fig 5:**
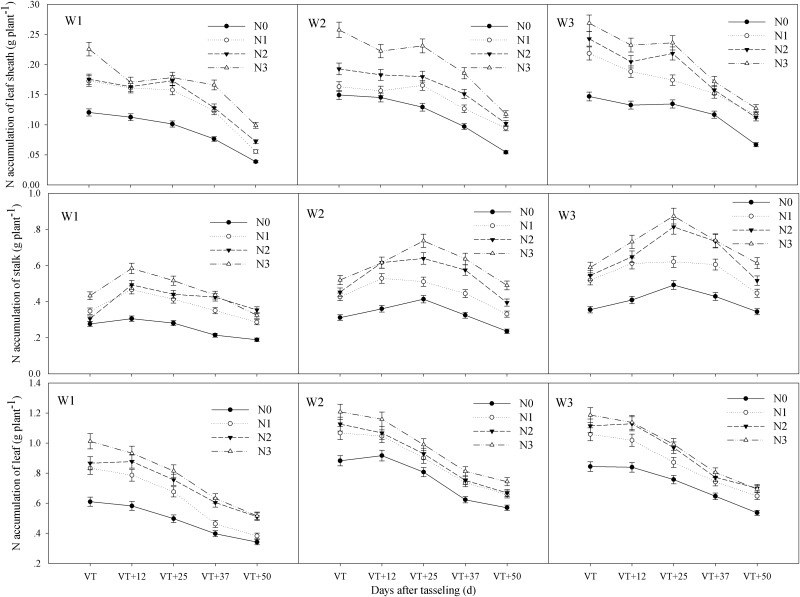
Changes of N content remaining in leaf sheath, stalk and leaf during grain filling period in summer maize. W1: severe water stress; W2: mild water stress; W3: adequate water condition; N0: no nitrogen; N1: N application of 105 kg hm^-2^; N2: N application of 210 kg hm^-2^; N3: N application of 315 kg hm^-2^; VT: tasseling stage.

The interactive effects of water and CRU on total N accumulation and N accumulation in bract, grain, leaf sheath, stalk, and leaf at different stages after tasseling occurred at an extremely significant level (P < 0.01) (Tables 4–6). The accumulation of N occurred over the whole growth period of summer maize and reached a maximum at maturity (Fig 3). N accumulation in the bract increased from 12 to 25 days after tasseling and then decreased to 50 days after tasseling, reaching its highest point at 25 days after the tasseling stage. Grain N accumulation increased continuously and slowly in the early stages and quickly in the later stages (Fig 4). N accumulation in the leaf sheath and leaf showed a gradual decline, decreasing relatively slowly in the early stage and quickly in the later stages. However, stalk N accumulation in W1 increased from tasseling to 12 days after tasseling and then decreased to maturity, but Stalk N accumulation in W2 and W3 increased from tasseling to 25 days after tasseling and then decreased to maturity (Fig 5). Total N accumulation increased with increasing amounts of CRU under the same water conditions. Compared with different water treatments, overall performance was W3 > W2 > W1. The total N accumulation of W2N3, W3N2 and W3N3 combinations were significantly higher than the other treatments at maturity (Fig 3).

**Table 4:** Analysis of variance for controlled release urea and water on total plant N accumulation of summer maize after tasseling stages (VT).

Average		Days after tasseling (d)			
	VT	VT+12	VT+25	VT+37	VT+50
W1	1.34±0.27c	1.57±0.36c	1.64±0.38c	1.91±0.43c	2.15±0.46c
W2	1.63±0.29b	2.03±0.37b	2.20±0.45b	2.33±0.53b	2.60±0.57b
W3	1.86±0.35a	2.29±0.51a	2.56±0.57a	2.78±0.49a	2.99±0.55a
ANOVA					
W	**	**	**	**	**
N	*	**	**	**	**
W×N	**	**	**	**	**

* and ** indicate significant difference at the 0.05 and 0.01 levels of probability, respectively.

**Table 5:** Analysis of variance for controlled release urea and water on N accumulation in bract and grain of summer maize after tasseling stages (VT).

Average	Bract				Grain			
	Days after tasseling (d)							
	VT+12	VT+25	VT+37	VT+50	VT+12	VT+25	VT+37	VT+50
W1	0.05±0.02b	0.12±0.01c	0.09±0.02c	0.06±0.01c	0.11±0.05b	0.28±0.10b	0.81±0.16c	1.30±0.28c
W2	0.05±0.02b	0.17±0.06b	0.16±0.07b	0.11±0.05b	0.29±0.09a	0.45±0.13a	0.92±0.20b	1.51±0.31b
W3	0.07±0.03a	0.24±0.07a	0.22±0.03a	0.17±0.04a	0.32±0.14a	0.50±0.14a	1.07±0.21a	1.65±0.28a
ANOVA								
W	*	**	**	**	**	**	**	**
N	*	*	*	NS	*	**	**	**
W×N	**	**	**	**	**	**	**	**

NS means not significant,

* and ** indicate significant difference at the 0.05 and 0.01 levels of probability, respectively.

**Table 6:** Analysis of variance for controlled release urea and water on N accumulation in leaf sheath, stalk and leaf of summer maize after tasseling stages (VT).

	Average	Days after tasseling (d)				
		VT	VT+12	VT+25	VT+37	VT+50
Leaf sheath	W1	0.17±0.04b	0.15±0.03b	0.15±0.04b	0.12±0.04a	0.07±0.03a
	W2	0.19±0.05a	0.18±0.03a	0.18±0.04a	0.14±0.04a	0.09±0.03a
	W3	0.22±0.05a	0.19±0.04a	0.19±0.05a	0.15±0.02a	0.11±0.03a
	ANOVA					
	W	*	**	**	NS	NS
	N	NS	*	**	**	**
	W×N	*	**	**	*	*
Stalk	W1	0.34±0.07b	0.46±0.12b	0.41±0.10c	0.36±0.10c	0.29±0.07c
	W2	0.43±0.09a	0.53±0.12a	0.57±0.14b	0.50±0.14b	0.36±0.11b
	W3	0.50±0.10a	0.60±0.14a	0.70±0.18a	0.63±0.15a	0.48±0.11a
	ANOVA					
	W	**	**	**	**	**
	N	NS	*	**	**	**
	W×N	**	**	**	*	*
Leaf	W1	0.83±0.17b	0.79±0.15b	0.69±0.14c	0.52±0.11c	0.44±0.09b
	W2	1.02±0.16a	0.99±0.12a	0.82±0.09b	0.62±0.09b	0.54±0.08a
	W3	1.13±0.20a	1.11±0.18a	0.93±0.14a	0.72±0.09a	0.59±0.10a
	ANOVA					
	W	**	**	**	**	*
	N	NS	*	**	**	**
	W×N	*	*	**	**	*

NS means not significant,

* and ** indicate significant difference at the 0.05 and 0.01 levels of probability, respectively.

Under W1, the maximum N accumulation amounts in the bract and grain from each controlled release urea treatment were very low; the N uptakes of the stalks reached a maximum at 12 days after the tasseling stage and then decreased linearly. Under W2, the maximum N amounts of the bract and grain in N3 were significantly higher and this difference was more significant during the later stages; N accumulation in the stalks reached a maximum at 25 days after tasseling. Under W3, the maximum N amounts in the bract, grain, leaf sheath, stalk, and leaf in N2 were slightly lower than in N3, and they were both significantly higher than under the other treatments (Figs 3–5). Due to the effects of interaction between water and CRU, N accumulation in different organs showed clear differences (Tables 4–6).

### N uptake and utilization efficiencies

Final N uptakes and utilization efficiencies in summer maize were significantly influenced by different water and CRU combinations. A significance test showed that the effects of the interaction on NHI, NIE, ANUE, RE_N_, PNUE, and SNDR reached a highly significant level (Table 7). The NHI response to watering treatments (W1 > W2 > W3) was the reverse of the grain yield response to watering treatments (W3 > W2 > W1) (Tables 1 and 7). Under the same water conditions, both NHI and NIE decreased with a higher N application rate. Under W1 and W3 conditions, ANUE, RE_N_, PNUE, and SNDR responses to N were ranked N1 > N2 > N3 and these differences were consistently significant. However, for ANUE, RE_N_, and SNDR under W2, rankings were also N1 > N3 > N2, while for PNUE rankings were N3 > N1 > N2 (Table 7). The lower SNDR with an increase in N application suggested that plants were able to absorb greater amounts of N from fertilizer. Under W3, the ANUE, RE_N_, and PNUE of N2 were 54.2, 34.9, and 14.4% higher than N3, respectively, but the difference in yield (Table 1) was not significant. Treatments of W2N3 and W3N2 combinations maintained high N efficiency in combination with high yield performance.

**Table 7:** Impact of controlled release urea (N0: no nitrogen, N1: N application of 105 kg hm^-2^, N2: N application of 210 kg hm^-2^, N3: N application of 315 kg hm^-2^) on nitrogen harvest index (NHI), nitrogen internal efficiency (NIE), agronomic nitrogen use efficiency (ANUE), apparent recovery efficiency of applied nitrogen (RE_N_), physiological nitrogen use efficiency (PNUE), and soil nitrogen dependency ratio (SNDR) of summer maize under different water conditions (W1: severe water stress, W2: mild water stress, W3: adequate water condition).

Water	Nitrogen	NHI (%)	NIE (kg kg^-1^)	ANUE (kg kg^-1^ N)	RE_N_ (%)	PNUE (kg kg^-1^ N)	SNDR (%)
W1	N0	68.0±0.49a	49.9±1.23b				
	N1	69.3±0.83a	41.9±1.04c	5.8±0.31d	30.8±0.60b	18.9±0.44d	74.0±0.13a
	N2	65.4±0.55b	36.9±1.15d	3.3±0.15f	24.3±0.60d	13.5±0.20e	64.4±0.32b
	N3	69.3±0.67a	35.8±0.44d	3.1±0.25f	20.2±0.46e	15.3±0.38e	59.3±0.32c
W2	N0	66.1±0.71b	55.9±0.85a				
	N1	65.8±0.68b	47.5±0.48b	8.3±0.35b	36.6±0.65a	22.7±1.01bc	74.5±0.58a
	N2	65.1±0.72bc	44.3±0.76c	5.3±0.20de	26.0±0.46c	20.2±0.34c	67.3±0.45b
	N3	63.8±0.58c	42.7±0.64c	6.5±0.64c	26.2±0.25c	24.7±0.76b	57.7±0.57c
W3	N0	63.9±0.60c	52.3±1.12a				
	N1	61.0±0.60d	47.6±0.86b	9.7±0.44a	33.5±0.65a	29.1±0.66a	79.5±0.29a
	N2	62.8±0.60d	43.2±1.13c	7.4±0.45bc	30.9±0.32b	24.0±0.45b	67.7±0.37b
	N3	60.6±0.64d	41.4±0.61c	4.8±0.45e	22.9±0.50de	20.9±0.37c	65.3±0.37b
Average							
W1		68.1±1.83a	41.2±6.43b	4.1±1.50b	25.1±5.35a	15.9±b	65.9±7.51b
W2		65.2±1.05b	47.6±5.90a	6.7±1.51a	29.6±6.06a	22.6±a	66.5±8.44b
W3		62.1±1.56c	46.1±4.88a	7.3±2.45a	29.1±5.52a	24.7±a	70.8±4.56a
ANOVA							
W		**	**	*	NS	*	NS
N		NS	**	*	*	NS	**
W×N		**	**	**	**	**	**

In each data area, different letters within the same column indicate significant difference among treatments at *P*<0.05. NS means not significant,

* and ** indicate significant difference at the 0.05and 0.01 levels of probability, respectively.

## Discussion

### Yield and dry matter accumulation

It was well known that final dry matter accumulation of summer maize is highly responsive to different water conditions [35, 36]. This study found that dry matter accumulation of W3 was significantly higher compared to W2 and W1 (Fig 1 and Table 1). The reason for this is that W3 offered sufficient moisture to effectively promote aboveground growth of summer maize, which ensured the formation of high yield potential via increased grain numbers and weight (Table 1). Higher moisture availability also played a role in increasing the sink and source of N in W3. Gheysari et al. [24] demonstrated that the effects of N fertilizer on total aboveground biomass depended on the availability of water in the soil. The stunted plant growth under W1 and W2 treatments due to drought stress had adverse effects on dry matter accumulation. However, the dry matter accumulation of N3 did not decrease under mild water stress (W2) and showed no differences under W3N2 and W3N3 treatments in this study, which indicated that increasing controlled release urea (CRU) had a greater effect on alleviating water stress (Fig 1). Previous studies have shown that N fertilization could significantly increase dry matter accumulation in maize plants [37–39]. Zhao et al. [12] demonstrated that controlled release fertilizers could significantly increase aboveground dry matter accumulation in maize compared to common compound fertilizer. In our study, it increased with increasing amounts of CRU under the same water conditions, but there was no significant difference between N2 and N3 under adequate conditions, which suggested that N3 was excessive under W3.

Dry matter was the material basis for grain yield, and the first step was to increase dry matter production and create as much as possible [40]. The distribution of dry matter accumulation in different organs changed with the growth center, and that of the grain increased rapidly after tasseling. Ciampitti et al. [41] demonstrated that N fertilization could significantly promote the remobilization of nutrients from dry matter to the grain between the vegetative-stage and the reproductive-stage. In our study, the results suggested that dry matter accumulation under W3 was significantly higher than that under W2 and W1 with the same CRU (Fig 1). Under severe water stress, grain dry weight did not increase with the increase of CRU. Grain dry matter accumulation increased with higher amounts of CRU under mild water stress. When the water was adequate, the CRU of N2 could maximize grain dry weight (Fig 2). Those of W3N2 and W2N3 combinations were significantly higher in each sampling period during grain filling, which indicated that they were more favorable for the accumulation of dry matter. The interactive effects of water and CRU on dry matter accumulation and grain dry weight in summer maize reached a significant level (Tables 2 and 3). The reason was that managing for optimum interactions of water and CRU could effectively improve the photosynthetic capacity of ear leaves in summer maize [42], as well as improve the plant dry matter production capacity, promote the production and distribution of photosynthetic products to grain, and optimize high yields of summer maize.

### Plant N uptake relationships to N utilization efficiencies

Water and N management were known to be closely related with N translocation and partitioning in previous maize studies [23, 27, 43]. Miao et al. [22] reported that moderate water conditions were required from the filling stage to maturity to ensure the absorption and utilization of N in the soil and that this condition could keep the stalk and sheath N accumulation high. This condition also increased leaf N accumulation, which further improved the N accumulation in the ears and improved the utilization rate of N [22]. These findings are consistent with the results of this experiment. This study suggested that N accumulation and distribution were tightly coupled with water and CRU management (Tables 4–6). Under W1, the N uptake of each treatment reached a maximum at 12 days after tasseling stage and then decreased linearly to maturity. N accumulation in stalk reached a maximum at 25 days after tasseling under W2 and W3 conditions (Figs 4 and 5), which indicated that water was conducive to the accumulation of N in the stalk, which could provide more nutrition for growth during the grain filling stage. Due to the effects of the interaction between water and N, N accumulation in different organs of each treatment showed clear differences. Under severe water stress, the CRU release capacity was restricted under low moisture conditions, possibly because the mineral N availability and root activity in the soil were inhibited [44]. This resulted in the N participating in morphogenesis during the vegetative period (wherein plant growth was inhibited, thereby affecting the construction of N sinks and the accumulation of total N amounts. Thus, the results of N distribution and poor translocation seriously affected the N utilization, and RE_N_ was reduced with an increase in N application rate (Table 7). Under mild water stress, soil moisture limitation also markedly reduced N uptake causing more fertilizer to be left in the soil instead of being recovered by the crop [14]. An appropriate increase of CRU made N release capacity relatively high and this played a compensatory role in the reduction of N distribution and translocation caused by mild water stress and improved N utilization rates; the NIE showed no significant difference between N2 and N3. Under adequate water conditions, ANUE, PNUE, and RE_N_ under N2 were significantly higher than under N3 (Table 7). Interactions between water and N could promote N absorption, translocation, and distribution, and this information is based on results from previous studies [25, 27, 45]. A coordinated relationship between water and N may be needed to meet the growth and development needs of summer maize in order to improve N accumulation in different organs, and then promote accumulation in the ears. N uptake and translocation displayed a certain compensatory role under W1 and W2 conditions, as well as a promoter role under W3 conditions [46]. The compensatory role under W2 was more effective than that of W1. These results indicated that N2 could meet the requirement of maize growth and development under adequate water conditions, and that the CRU of N3 was too high and the NUE was low. N uptake and utilization were seriously affected by severe water stress and the RE_N_ was low. An increase of CRU under mild water stress may safeguard the nutrient supply in summer maize and improve RE_N_. Under adequate water conditions, appropriate application amounts of CRU were favorable to the interactions between water and N, promoting the growth and development of summer maize plants and improving N utilization rates (Table 7).

Previous studies have demonstrated that yields increase with increasing N application under the same water conditions; they also increase with increasing water content at the same N level [27, 42]. Shao et al. [44] found a significant positive coupling effect between N and water on maize yield. In our study, there were significant interactions between water and CRU on increasing maize yield and improving N utilization rates. Under severe water stress, the photosynthetic capacity of the ear leaf decreased as did the dry matter production capacity [42], which resulted in yield decreases and limited plant N uptake, which in turn seriously affected N utilization in the plant. An appropriate increase in CRU alleviated the mild water stress and yield increased with plant N uptake. Also, the yield of N3 under mild water stress showed no significant differences with the treatments of N2 and N3 under normal water conditions, indicating that the interactive effects of water and CRU in W2N3 effectively alleviated the mild water stress. The results showed that the N amount of N3 under adequate water conditions might be excessive, consistent with the results of Zhao et al. [12, 30] in their study on controlled release fertilizers. This is due to the interactive effects between water and CRU (Table 1), because higher water availability helped maintain concentrations of N levels during the whole growth period and ensured consistent N supply.

Additionally, CRU migration mechanisms in the soil under different water conditions influenced the maize yield and this process should be further investigated. We used pot conditions with a rainout shelter to precisely control water status during the whole growth period. But work of this nature should be repeated using field experiments and should consider different plant densities.

## Conclusions

Controlled release urea and water had significant interactive impacts on N absorption, distribution, and efficiency in summer maize. Managing for optimum interactions of N and soil moisture was conducive to high yields, improved N accumulation of summer maize during the grain filling stage, and coordinated N distribution and accelerated N translocation to the ears. This approach could improve the N utilization rate and increase the dry matter accumulation in summer maize, especially the yield components of grains per ear and 1000-grain weight. This study found that the controlled release urea application rate of 210 kg N ha^–1^ was the best treatment when the soil moisture content was maintained at 75% ± 5% of the field capacity. We suggest that a higher rate of 315 kg ha^–1^ is more justifiable in semi-arid regions where soil moisture content is maintained at 55% ± 5% of the field capacity.

## References

[1] Li YJ (2013) Present situation and Prospect of China's corn import trade. Agricultural Outlook 07(6): 47–50.

[2] Chen XP, Cui ZL, Fan MS, Peter V, Zhao M, Ma WQ, et al (2014) Producing more grain with lower environmental costs. Nature 514(7523): 486–489. doi: 10.1038/nature13609 25186728 10.1038/nature13609

[3] Tilman D, Cassman KG, Matson PA, Naylor R, Polasky S (2002) Agricultural sustainability and intensive production practices. Nature 418(6898): 671–677. doi: 10.1038/nature01014 12167873 10.1038/nature01014

[4] Ge T, Sui F, Bai L, Tong C, Sun N (2012) Effects of water stress on growth, biomass partitioning and water use efficiency in summer maize (*Zea mays* L.) throughout the growth cycle. Acta Physiologiae Plantarum 34(3): 1043–1053.

[5] Zhang SL, Sadras V, Chen XP, Zhang FS (2014) Water use efficiency of dryland maize in the Loess Plateau of China in response to crop management. Field Crops Research 163(1): 55–63.

[6] Duvick DN (1992) Genetic contributions to advances in yield in U.S. maize. Maydica 37: 69–79.

[7] Tollenaar M, Lee EA (2002) Yield potential, yield stability and stress tolerance in maize. Field Crops Research 75: 161–169.

[8] Zhao RF, Chen XP, Zhang F S (2009) Nitrogen cycling and balance in winter-wheat-summer-maize rotation system in northern china plain. Acta Pedologica Sinica 46(4): 684–697.

[9] Zhang W, Cao G, Li X, Zhang H, Wang C, Liu Q, et al (2016) Closing yield gaps in China by empowering smallholder farmers. Nature 537(7622): 671–674. doi: 10.1038/nature19368 27602513 10.1038/nature19368

[10] Lal R (2013) Climate-strategic agriculture and the water-soil-waste nexus. Journal of Plant Nutrition and Soil Science 176(4): 479–493.

[11] Paponov IA, Engels C (2005) Effect of nitrogen supply on carbon and nitrogen partitioning after flowering in maize. Journal of Plant Nutrition & Soil Science 168(4): 447–453.

[12] Zhao B, Dong ST, Zhang JW, Liu P (2013) Effects of controlled-release fertilizer on nitrogen use efficiency in summer maize. PLOS ONE 8(8): e70569 doi: 10.1371/journal.pone.0070569 23936449 10.1371/journal.pone.0070569 PMC3732217

[13] Duan Y, Xu M, Wang B, Yang X, Huang S, Gao S (2011) Long-term evaluation of manure application on maize yield and nitrogen use efficiency in china. Soil Science Society of America Journal 75(4): 1562.

[14] Teixeira EI, George M, Herreman T, Brown H, Fletcher A, Chakwizira E, et al (2014) The impact of water and nitrogen limitation on maize biomass and resource-use efficiencies for radiation, water and nitrogen. Field Crops Research 168: 109–118.

[15] Zhang M, Shi YX, Yang SX, Yang YC (2001) Status quo of study of controlled-release and slow-release fertilizers and progress made in this respect. Journal of Chemical Fertilizer Industry 28(5): 27–30.

[16] Wu ZJ, Zhou JM (2001) Present situation, trend and strategy of control-released fertilizer and slow-released fertilizer in China. Review of China Agricultural Science and Technology 3(1): 73–76.

[17] Shaviv A (2001) Advances in controlled-release fertilizers. Advances in Agronomy 71(01): 1–49.

[18] Diez JA, Caballero R, Bustos A, Roman R, Cartagema MC, Vallejo A (1994) Control of nitrate pollution by application of controlled release fertilizer (CRF), compost and an optimized irrigation system. Nutrient Cycling in Agroecosystems 43(1): 191–195.

[19] Wei L, Ma C, Huang XS, Du YY, Wang TZ (2010) Effects of controlled-release nitrogen fertilizer on carbon and nitrogen metabolism of summer maize. Plant Nutrition and Fertilizer Science 16(3): 773–776.

[20] Hu H, Ning T, Li Z, Han H, Zhang Z, Qin S, et al (2013) Coupling effects of urea types and subsoiling on nitrogen–water use and yield of different varieties of maize in northern China. Field Crops Research 142(1): 85–94.

[21] Nannen DU, Herrmann A, Loges R, Dittert K, Taube F (2011) Recovery of mineral fertiliser N and slurry N in continuous silage maize using the ^15^N and difference methods. Nutrient Cycling in Agroecosystems 89(2): 269–280.

[22] Miao WF, Chen SY, Shao LW, Sun HY, Zhang XY (2011) Effect of irrigation on nitrogen uptake and translocation in summer maize. Chinese Journal of Eco-Agriculture 19(2): 293–299.

[23] Huang P, Zhang J, Zhu A, Xin X, Zhang C, Ma D, et al (2014) Coupled water and nitrogen (N) management as a key strategy for the mitigation of gaseous N losses in the Huang-Huai-Hai Plain. Biology & Fertility of Soils 51(3): 1–10.

[24] Gheysari M, Mirlatifi SM, Bannayan M, Homaee M, Hoogenboom G (2009) Interaction of water and nitrogen on maize grown for silage. Agricultural Water Management 96(5): 809–821.

[25] Jia XC, Shao LJ, Liu P, Zhao BQ, Gu LM, Dong ST, et al (2014) Effect of different nitrogen and irrigation treatments on yield and nitrate leaching of summer maize (*Zea mays* L.) under lysimeter conditions. Agricultural Water Management 137(1385): 92–103.

[26] Bahman E, Maranville JW (1991) Interactive effects of water and nitrogen stresses on nitrogen utilization efficiency, leaf water status and yield of corn genotypes. Communications in Soil Science and Plant Analysis 22(13–14): 1367–1382.

[27] Guo LW, Ning TY, Nie LP, Li ZJ, Lai R (2016) Interaction of deep placed controlled-release urea and water retention agent on nitrogen and water use and maize yield. European Journal of Agronomy 75: 118–129.

[28] Lu RK (2000) Soil and agricultural chemistry analysis method. China Agricultural Science and Technology Press, Beijing, China.

[29] Jiang PF, Lei TW, Liu XH, Wu Y, Li X, Wang QJ (2006) Principles and experimental verification of capillary suction method for fast measurement of field capacity. Transactions of the Chinese Society of Agricultural Engineering 22(7): 1–5.

[30] Zhao B, Dong ST, Zhang JW, Liu P (2010) Effects of controlled-release fertilizer on yield and nitrogen accumulation and distribution in summer maize. Acta Agronomica Sinica 36(10): 1760–1768.

[31] Geng J, Chen J, Sun Y, Zheng W, Tian X, Yang Y, et al (2016) Controlled release urea improved nitrogen use efficiency and yield of wheat and corn. Agronomy Journal 108(4): 1–8.

[32] Ciampitti IA., Vyn TJ(2013) Grain nitrogen source changes over time in maize: A Review. Crop Science 53(2): 366–377.

[33] Moll RH, Kamprath EJ, Jackson WA (1982) Analysis and interpretation of factors which contribute to efficiency of nitrogen utilization. Agronomy Journal 74: 562–564.

[34] Huggins DR, Pan WL (1993) Nitrogen Efficiency Component Analysis: An Evaluation of Cropping System Differences in Productivity. Agronomy Journal 85(4): 898–905.

[35] Frederick JR., Below FE, Hesketh JD (1990) Carbohydrate, nitrogen and dry matter accumulation and partitioning of maize hybrids under drought stress. Annals of Botany 66(4): 407–415.

[36] Fang Q, Ma L, Yu Q, Malone RW, Saseendran SA, Ahuja LR (2008) Modeling nitrogen and water management effects in a wheat-maize double-cropping system. Journal of Environmental Quality 37(6): 2232–2242. doi: 10.2134/jeq2007.0601 18948476 10.2134/jeq2007.0601

[37] Mueller SM, Vyn TJ (2016) Maize plant resilience to N stress and post-silking N capacity changes over time: A Review. Frontiers in Plant Science 7(7). 10.3389/fpls.2016.00053 PMC4746326 26904038

[38] Chen K, Kumudini SV, Tollenaar M, Vyn TJ (2015) Plant biomass and nitrogen partitioning changes between silking and maturity in newer versus older maize hybrids. Field Crops Research 183: 315–328.

[39] Parija B, Kumar M (2013) Dry matter partitioning and grain yield potential of maize (*Zea mays* L.) under different levels of farmyard manure and nitrogen. Journal of Plant Science Research 29(2): p177.

[40] Allison JCS, Watson DJ (1966) The production and distribution of dry matter in maize after flowering. Annals of Botany 30(3): 365–381.

[41] Ciampitti IA, Zhang H, Friedemann P, Vyn TJ (2012) Potential physiological frameworks for mid-season field phenotyping of final plant nitrogen uptake, nitrogen use efficiency, and grain yield in maize. Crop Science 52(6): 2728–2742.

[42] Li GH, Zhao B, Dong ST, Liu P, Zhang JW, He ZJ (2015) Effects of coupling controlled release urea with water on yield and photosynthetic characteristics in summer maize. Acta Agronomica Sinica 41(9): 1406–1415.

[43] Ciampitti IA, Vyn TJ (2011) A comprehensive study of plant density consequences on nitrogen uptake dynamics of maize plants from vegetative to reproductive stages. Field Crops Research 121(1): 2–18.

[44] Shao GQ, Li ZJ, Ning TY, Zheng YH (2013) Responses of photosynthesis, chlorophyll fluorescence, and grain yield of maize to controlled-release urea and irrigation after anthesis. Journal of Plant Nutrition and Soil Science 176(4): 595–602.

[45] Gheysari M, Mirlatifi SM, Homaee M, Asadi ME, Hoogenboom G (2009) Nitrate leaching in a silage maize field under different irrigation and nitrogen fertilizer rates. Agricultural Water Management 96(6): 946–954.

[46] Peng Y, Sun YJ, Jiang MJ, Xu H, Qin J, Yang ZY, et al (2014) Effects of water management and slow/controlled release nitrogen fertilizer on biomass and nitrogen accumulation, translocation, and distribution in rice. Acta Agronmica Sinica 40(5): 859–870.

